# Gastric Cancer (Biomarkers) Knowledgebase (GCBKB): A Curated and Fully Integrated Knowledgebase of Putative Biomarkers Related to Gastric Cancer

**Published:** 2007-02-07

**Authors:** Bernett T. K. Lee, Chun Meng Song, Boon Huat Yeo, Cheuk Wang Chung, Ying Leong Chan, Teng Ting Lim, Yen Bing Chua, Marie C. S. Loh, Boon Keong Ang, Praveen Vijayakumar, Lailing Liew, Jiahao Lim, Yun Ping Lim, Chee Hong Wong, Danny Chuon, Gunaretnam Rajagopal, Jeffrey Hill

**Affiliations:** Bioinformatics Institute, 30 Biopolis Street, #07–01 Matrix, Singapore 138671, Singapore

## Abstract

The Gastric Cancer (Biomarkers) Knowledgebase (GCBKB) (http://biomarkers.bii.a-star.edu.sg/background/gastricCancerBiomarkersKb.php) is a curated and fully integrated knowledgebase that provides data relating to putative biomarkers that may be used in the diagnosis and prognosis of gastric cancer. It is freely available to all users. The data contained in the knowledgebase was derived from a large literature source and the putative biomarkers therein have been annotated with data from the public domain. The knowledgebase is maintained by a curation team who update the data from a defined source. As well as mining data from the literature, the knowledgebase will also be populated with unpublished experimental data from investigators working in the gastric cancer biomarker discovery field. Users can perform searches to identify potential markers defined by experiment type, tissue type and disease state. Search results may be saved, manipulated and retrieved at a later date. As far as the authors are aware this is the first open access database dedicated to the discovery and investigation of gastric cancer biomarkers.

## Introduction

Although the overall incidence of gastric cancer worldwide is decreasing, it is still a major health problem and, after lung cancer, it is the second leading cause of cancer death (Hohenberger and Gretschel, 2003). It has been estimated that there are 876 000 new cases diagnosed and 649 000 deaths reported per annum worldwide (Roberts-Thompson and Butler, 2005). Countries of high incidence include Singapore, China, Japan and Russia (Look et al. 2001). The overall decrease in the incidence of gastric cancer has been attributed to domestic refrigeration, a decrease in the intake of salted, smoked and pickled foods and the greater availability and consumption of fresh fruit and vegetables. There are other environmental factors, apart from diet, that increase the risk of gastric cancer in susceptible individuals including tobacco smoking (Nouraie et al. 2005), alcohol consumption (Leung et al. 2005) and *Helicobacter pylori* infection (Normark et al. 2003). A two to three fold increase in the risk of gastric cancer has been linked to *H. pylori* infection in some but not all populations (Look et al. 2001). There is epidemiological evidence of genetic risk factors associated with gastric cancer including higher than predicted concordance rates in identical and non-identical twins and first degree relatives of patients with gastric cancer having a two to three fold increased risk of developing the disease (El-Omar et al. 2000).

Gastric carcinoma is characterized by aggressive metastasis. There are a number of molecular factors that are thought to contribute to this property including microsatellite instability, activation of oncogenes, inactivation of tumor suppressor genes and abnormal activation of telomerase (Wong et al. 2006). These factors have an enormous influence on the control of cell growth and differentiation and give rise to very resilient gastric cancer cells. The aggressive metastasis and the high resilience of the cancerous cells contribute to the very poor survival rates of gastric cancer patients. The 5 year survival rate in patients who undergo surgery is less than 40% (Wanebo et al. 1993). However, survival rates are vastly improved when gastric cancer is diagnosed early. Studies have shown that up to 80% of patients diagnosed with gastric cancer have advanced and incurable stage IV disease and only 1% have early disease (Sue-Ling, 1998). To improve the survival rates of this disease, earlier diagnosis is vital. In countries such as Japan, where the incidence of the disease is high, mass screening of the population has led to higher detection levels of the disease at an earlier stage. However, in other populations, screening of high risk individuals is thought to be economically more viable. Those considered to be at risk include patients with symptoms of pernicious anemia, gastric ulcer and chronic atrophic gastritis. The main form of screening involves the use of the two procedures, endoscopy and biopsy. The invasive nature of these two techniques makes routine screening for gastric cancer both undesirable for the patient and expensive for the health care provider. This has provided the impetus for researchers to focus their efforts on the discovery of diagnostic biomarkers that can be detected in readily accessible body fluids (for example serum, plasma and urine) for the early detection of gastric cancer.

Biomarkers can be defined as molecules that can be measured in a patient’s body fluids or tissues to diagnose a disease at the molecular level, to monitor the response to therapy or predict the prognosis for a given disease. In the past twenty years less than 12 biomarkers have been approved by the FDA for use in cancer diagnosis and therapy (Anderson and Anderson, 2002). However, literally thousands of putative biomarkers for cancer diagnosis and detection have been discovered and described in the literature (Manne et al. 2005). The generation of this wealth of data has been driven by the development of new techniques such as DNA microarrays, mass spectroscopy and tissue arrays. A not insignificant number of the putative cancer biomarkers reported in the literature are related to gastric cancer. This data is of great value to both scientific researchers and clinicians alike who are working in the field of gastric cancer biomarker discovery. In order to facilitate the discovery process, a project was initiated to construct an integrated user friendly knowledge-base that catalogues putative markers related to the gastric cancer disease process. It is hoped that this freely accessible resource will be equally as valuable to the experimentalist as it will be to the *in silico* researcher.

## Overview of the Database

The gastric cancer biomarker knowledgebase (GCBKB) is a web based resource that contains data on putative gene and protein biomarkers that have been identified as being associated with the gastric cancer process. The data is derived from peer reviewed journals and is updated on a regular basis by a curation team. The GCBKB also contains data from experimental collaborators working in the field of gastric cancer biomarker discovery. Data submitted to the curators of the knowledge-base will be reviewed by them and a panel of local experts. Once an agreement is reached between the collators, the experimentalists and the panel, the data will be uploaded to the knowledgebase.

The putative markers that can be found in the knowledgebase are annotated by KLEGO. KLEGO is a fully automated computational annotation pipeline that was developed by the Bioinformatics Institute, A-Star, Singapore. It extracts annotation data from the UCSC genome browser (Karolchik et al. 2003) and the UniProt (Bairoch et al. 2005), Gene Ontology (Ashburner et al. 2001), InterPro (Mulder et al. 2002), Pfam (Finn et al. 2006), BIND (Alfarano et al. 2005), IntAct (Hermjakob et al. 2004), KEGG (Kanehisa et al. 2004), PDB (Berman et al. 2003) and UniGene (Boguski and Schuler, 1995) databases. The data is integrated and displayed on a standard web browser where visualization tools such as the chromosome viewer, the gene ontology viewer and the interaction viewer are available for users to visualize their data in a number of formats. KLEGO is freely available to all users and can be found at the following URL: http://klego.bii.a-star.edu.sg/.

The GCBKB allows users to perform searches based on selected criteria and provides the facility for them to save their search results. After saving sets of data, users are also able to manipulate those data sets by performing Boolean type procedures. The GCBKB will greatly enhance the analysis of putative gastric cancer biomarkers by the scientific community.

The technical details of the knowledge base are as follows:

Server: Sun Fire V240 running Solaris with 2 UltraSPARC IIIi processors each rated at 1GHz with 2GB of memory.

Web Server: Apache 2.0.58 running mod_ php5.

Database: MySQL 4.1.14

## Data Source

The initial data in the knowledgebase is derived from 130 research articles published in 60 peer reviewed journals between 1998 and 2005 and was purchased from Jubilant Biosys (http://www.jubilantbiosys.com/index.htm). The putative gene and protein biomarkers identified in those 130 journals have been annotated using KLEGO. Additional data is regularly added to the GCBKB from 7 journals:

- World Journal of Gastroenterology
- Clinical Cancer Research
- International Journal of Cancer
- Oncogene
- Cancer Research
- Gastric Cancer
- Proteomics

There were a number of factors considered when the 7 journals were chosen including the journal source of the original 130 articles, the wish to obtain a balance of oncology, gastrointestinal and technology based publications and also the wishes of external collaborators. The curation team examines the 7 journals and, based on a series of selection criteria, extract articles relevant to gastric cancer biomarkers. The selection criteria stipulate that the putative biomarker be either a protein, an indicator of expression (i.e. an mRNA) or a single nucleotide polymorphism. There also has to be a demonstrable association between some aspect of the gastric cancer disease process and the putative marker identified. The data is considered of sufficient quality as it has gone through the peer review process of the journals in which it was originally published. The abstracts to those articles are uploaded to the knowledgebase and the putative biomarkers identified. The gene and protein biomarkers are then annotated using KLEGO. To date (October, 2006) there are details of 159 journal articles from which 443 putative gene and 85 putative protein gastric cancer biomarkers have been identified.

## Access and Use

Use of the GCBKB is open to all. However, for users to securely save their data, a simple registration procedure must be performed.

**Figure 1:**
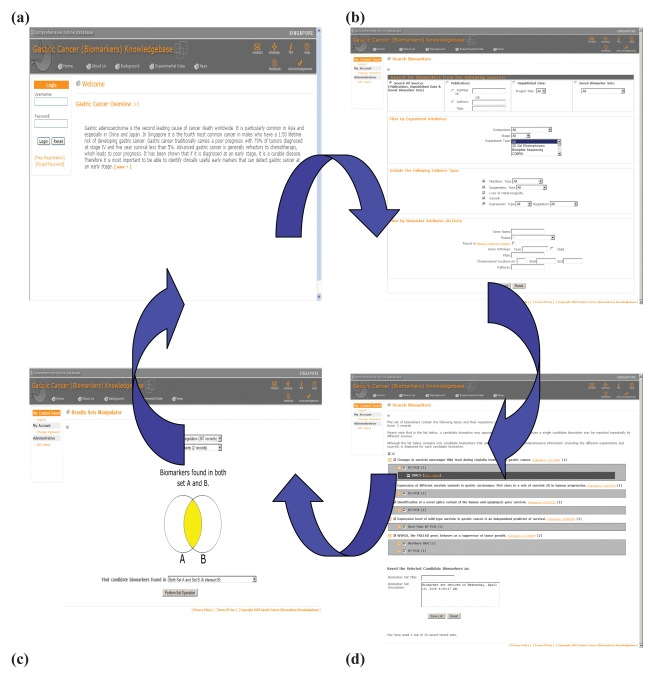
A screenshot of the GCBKB showing the (**a**) home page, (**b**) search page, (**c**) results page and (**d**) results set manipulator page.

From the home page (Figure. 1a) the user can access the search page (Figure. 1b) from where the search criteria may be selected. Searches may be carried out according to experiment type, comparison of tissue type (healthy versus disease) and stage of disease. This allows users to differentiate between putative biomarkers that are differentially expressed between healthy and cancerous tissue and putative biomarkers whose expression levels reflect disease progression. Further filters may be applied to searches for example whether there is evidence of mutation or whether the protein has been identified in plasma by the Plasma Proteome Project (http://www.bioinformatics.med.umich.edu/hupo/ppp). Results of searches are presented on a results page (Figure. 1c) which shows details of publications as well as the experimental details selected to perform the search. The page is collapsible/expandable to allow the user to determine how much information is displayed at any one time. If the PubMed ID is selected, details of the publication can be viewed with a list of experiments described in that publication. The experiment ID then allows the user to view details of the particular experiment and view a list of the putative biomarkers identified. Individual biomarkers may be selected to reveal more information and annotation by KLEGO. All or parts of the search results may be saved.

The results manager can be accessed from the experimental data menu which appears on all views. It allows users to perform editing functions on their saved data sets such as deletion, duplication and data sharing. Results can be visualised using the tools provided by KLEGO which is accessed via the KLEGO icon. The experimental data menu also contains a link to the results sets manipulator (Figure. 1d) which allows users to perform Boolean type operations on saved data sets. For example common putative biomarkers found in two different searches and saved in two separate data sets can be identified.

To extract meaningful information from datasets, they have to be interrogated by search criteria. To refine the results obtained and to make them more specific, further rounds of searching can be performed upon them with ever more stringent criteria. Users of the GCBKB are able to perform this type of iterative procedure and then perform Boolean operations such as union and intersect upon the generated search results.

**Figure 2:**
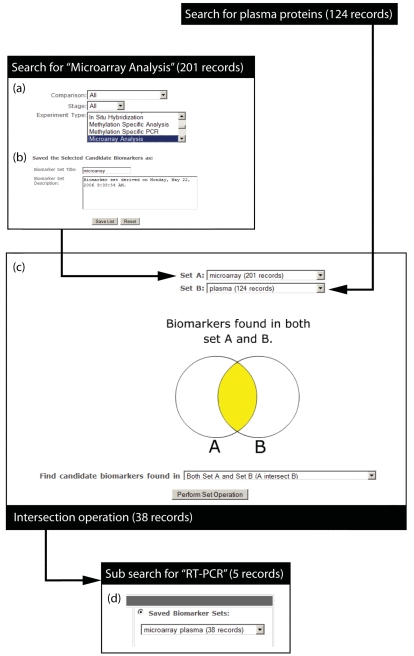
Schematic view of worked search example showing (**a**) selection of search criteria, (**b**) saving of data set, (**c**) intersect operation and (**d**) final filtering of data.

To demonstrate one of the many applications of the GCBKB, a theoretical biomarker search is outlined. An investigator is interested in studying putative biomarkers of gastric cancer identified by transcriptomic analyses. If the database is interrogated using ‘microarray analysis’ as the main search criteria (Figure. 2a), a list of 201 genes, that show differential expression in gastric cancer, is generated. The set of results may be saved and entitled ‘microarray’ (Figure. 2b). The investigator requires that the putative biomarkers be measured in plasma. The database can be interrogated for putative biomarkers identified in plasma by checking the ‘found in plasma proteome project’ box in the section ‘filter by biomarker attributes (KLEGO)’ on the search page. This results in 99 genes and 25 proteins being identified and may be saved as data set ‘plasma.’ To determine which of the 201 genes identified by microarray have been identified in plasma the ‘manipulate results’ tool can be used to identify genes common to both sets of saved data by performing an intersection operation (Fig. 2c). This results in the identification of 38 candidate biomarkers genes which may be saved as set ‘microarray plasma.’ The investigator then wishes to validate the transcriptomic data by filtering with the ‘RT-PCR’ experiment type criteria to determine which genes with differential expression have been verified by RT-PCR (Fig. 2d). The investigator is finally presented with 5 putative biomarkers whose genes are differentially expressed in gastric cancer, whose protein products have been identified in plasma and whose expression changes have been verified by RT-PCR. The resulting 5 genes (MMP2, CCKBR, TIMP1, DAPK1, CAPN9) are presented to the user in such a way that abstracts of publications and details of experimental data are readily accessible.

## Future Direction

In order to provide a greater coverage of technology based developments that are pertinent to the field of gastric cancer biomarker discovery, the number of source journals (currently standing at 7) will be expanded to include journals with more proteomic and transcriptomic content. In order to overcome any biases that may be generated in the knowledgebase, due to the selection of journals that are curated, work has started on the development of a text mining engine which will be able to search open access databases, such as PubMed, for relevant articles. Identified publications will be manually curated, as they are at present, and the relevant biomarker data uploaded to the knowledgebase. This will greatly expand the data content of the database with out greatly increasing the resources required to maintain it.

Over the past few years, the field of biomarker discovery has benefited from a considerable increase in expenditure on research and development in both the academic and industrial sectors. Recent scientific drivers of this trend include the sequencing of the human genome, the detailed comparisons of diseased versus healthy transcriptomes and the advances made in analyzing the complexities of the human proteome. Regulatory policy making decisions are also contributing to the increased activity; the U.S. FDA has identified biomarkers as crucial components in the Critical Path Initiative for drugs and diagnostics (Phillips et al. 2006). This federally funded initiative has been instigated in an attempt to explain how increased research and development expenditure by the pharmaceutical industry has not led to an increase in new drug and diagnostic products being made available to patients. The increase in research in the biomarker field is resulting in the generation of ever increasing amounts of data and as a consequence there is a need for more data storage devices and data analysis tools. More knowledgebases, such as the one described in this article, will be generated to catalogue putative biomarkers relevant to other diseases. This will result in a large dataset of proteins and genes which, with the necessary validation, will help researchers diagnose disease, predict response to therapy and foresee clinical outcomes. This dataset of putative gene and protein biomarkers will be dynamic with new members being added as more studies are performed. Putative biomarkers will also be removed from the dataset as they are subjected to more stringent validation procedures and their value as predictive entities decreases. This ever changing amalgam of genes and proteins can be thought of as the ‘predictome.’

To conclude, the primary objective of this project was to generate a knowledgebase that would be a valuable tool for all those interested in the discovery of gastric cancer biomarkers. Hopefully this has been achieved and the GCBKB will facilitate the discovery of diagnostic biomarkers to enable the early detection of gastric cancer. It may also help in the discovery biomarkers that can be used to monitor the progression of the disease, assess the efficacy of treatment and stratify the disease into subtypes. This in turn may lead to increased survival rates and the advent of more personalized treatments for patients.
